# Optimal surveillance against foot-and-mouth disease: A sample average approximation approach

**DOI:** 10.1371/journal.pone.0235969

**Published:** 2020-07-09

**Authors:** Tom Kompas, Pham Van Ha, Hoa-Thi-Minh Nguyen, Graeme Garner, Sharon Roche, Iain East

**Affiliations:** 1 Centre of Excellence for Biosecurity Risk Analysis, School of Biosciences and School of Ecosystem and Forest Sciences, University of Melbourne, Victoria, Australia; 2 Australian Centre for Biosecurity and Environmental Economics, Crawford School of Public Policy, Australian National University, Canberra, ACT, Australia; 3 Crawford School of Public Policy, Australian National University, Canberra, ACT, Australia; 4 Department of Agriculture, Animal Health Epidemiology, Canberra, ACT, Australia; Plum Island Animal Disease Center, UNITED STATES

## Abstract

Decisions surrounding the presence of infectious diseases are typically made in the face of considerable uncertainty. However, the development of models to guide these decisions has been substantially constrained by computational difficulty. This paper focuses on the case of finding the optimal level of surveillance against a highly infectious animal disease where time, space and randomness are fully considered. We apply the Sample Average Approximation approach to solve our problem, and to control model dimension, we propose the use of an infection tree model, in combination with sensible ‘tree-pruning’ and parallel processing techniques. Our proposed model and techniques are generally applicable to a number of disease types, but we demonstrate the approach by solving for optimal surveillance levels against foot-and-mouth disease using bulk milk testing as an active surveillance protocol, during an epidemic, among 42,279 farms, fully characterised by their location, livestock type and size, in the state of Victoria, Australia.

## 1 Introduction

Increasing globalisation and mobility has heightened the risk of bio-invasions by invasive alien species (IAS) and transboundary animal diseases (TAD) [1]. The damages caused by IAS and TAD to biodiversity and the economy are substantial [2, 3]. While prevention is the first line of defence, focusing on ports of entry, border quarantine and main pathways, complete prevention at the border (and through pre-border activities) has proven impossible. For this reason, a good deal of attention in the literature and in policy making has been paid recently to local or post-border surveillance, where there exists a trade-off between spending on surveillance against an IAS/TAD, at any point in time, and the cost of controlling its establishment and spread in the future [4, 5].

Methodologically, finding the optimal level of local surveillance against an IAS/TAD is an especially challenging task for two main reasons. First, an invasion is typically random in both time and space [6–8]. Second, its diffusion is highly dependent on local spatial characteristics [6, 9, 10]. These features make the search for optimal surveillance, or the best point of early detection, a difficult stochastic spatial dynamic optimisation problem which almost certainly faces the ‘curse of dimensionality’, or a computational impasse due to the excessive size or dimension of the model [11].

At the risk of oversimplification, there are four standard modelling approaches to aid decision-making in this class of biosecurity problems. The first is the aggregate approach which largely ignores or reduces the spatial dimension (e.g. [4, 12–15]). However, as Wilen [16] and Meentemeyer et al. [17] suggest, treating spatial heterogeneity in a (near) uniform manner in this way can produce misleading results. The second approach focuses on the spatial aspect of invasions, thus determining a one-time surveillance effort [18–20], or designing long-term equilibrium surveillance programs using steady-state analysis [21]. Recently, Epanchin-Niell and Wilen [22] proposed a third approach that explicitly and fully considered both time and space dimensions, but in a deterministic setting. Their model has been extended by Chalak et al. [23] to incorporate a limited range of stochasticity, but is only able to accommodate a small range of landscape heterogeneity (i.e., a 15-cell x 15-cell). This limited spatial heterogeneity is likely insufficient for most practical bio-invasion modelling exercises.

In parallel, some studies abandon optimization routines altogether to avoid the curse of dimensionality, and choose instead simulation methods to retain all of the features of time, space and randomness [9, 24–31]. However, the downside of these simulation methods is their inability to generate optimal solutions as only a small number of policy and disease transmission scenarios can normally be simulated.

A fourth approach, a simulation-based optimisation has also been proposed [32, 33]. Technically, this approach involves two stages. In the first stage, a detailed spread model is developed to simulate the development of the disease over both time and space in a random manner. Simulation outcomes are used to estimate the average trend of the invasion development or dispersal parameters. In the second stage, an optimisation model is solved using only the estimated parameters, thus facing no issues with dimensionality. Despite their important contribution, these models are not fully spatially explicit, and are thereby at risk of missing some important spatial features of an invasion during the estimation of transmission parameters.

In general terms, the approach to stochastic optimisation is highly dependent on the structure of a particular problem. Stochastic optimisation problems are generally classified based on the number of time periods at which decisions are made. The optimal surveillance problem, our focus, can be formulated as a two-stage problem, the most widely-used form in stochastic programming. At the first stage, a decision has to be made on how much to spend on early detection before a bio-invasion is realised in the second stage. Thus, the task boils down to minimising the sum of the active surveillance cost in the first stage and the expected damage caused by the invasion in the second stage. Given time and space dimensions, the number of probable invasion scenarios can be very large, making it impossible to solve the problem directly.

One solution method to this class of problems is the Sample Average Approximation (SAA) approach. It is arguably preferred over other more popular approaches, such as gradient approximations, because it does not impose any structure on the spread function—a requirement that has been criticised in recent literature (e.g. [34]). Technically, SAA is a two-part method that uses sampling and deterministic optimisation [35]. The sampling techniques help reduce the scenario set to a manageable size and accordingly find solution candidates by using sample averages. Deterministic optimisation is then used to search for the optimal solution.

SAA is best known for its simplicity and desirable asymptotic statistical properties. Estimates of global optima are guaranteed if the set of decisions is convex and the objective function is convex in policy choices for all scenarios. Nonetheless, the challenge that prevents its full application is computational expense, since scenarios are processed in a batch manner while solution quality depends on the sample size of scenarios. Therefore, while SAA has been applied in various fields, it often remains limited to problems of small size [36, 37]. In contrast, surveillance problems where time, space and randomness are explicitly and fully considered are prohibitively large in dimension, making simulations, let alone optimisation procedures, especially challenging [10].

Against this background, we aim to make two specific contributions to the surveillance literature on TADs. First, we circumvent computational complexity by: (a) designing an ‘infection tree model’ to capture infection paths, instead of the more typical approach of tracking farms and ‘contacts’ as in network models; and (b) using a combination of innovative ‘pruning’ of the infection tree and parallel processing methods. With this contribution, SAA becomes amenable to stochastic surveillance optimisation problems for TADs. In our paper, in short, we broaden this class of models to include problems that involve finding optimal control strategies in a stochastic setting, where the dimensions of space and time are fully specified, while avoiding a computational impasse. Second, we demonstrate our approach by solving for optimal surveillance against foot-and-mouth disease (FMD) using bulk milk testing (BMT), as an active surveillance protocol, across 42,279 farms, fully characterised by their location, livestock type and size, in Victoria, Australia.

## 2 Model formulation and methods

Without any policy interventions, an animal disease can be either ‘naturally immunised’ (i.e., assuming post-infection recovery) or detected by front-line people such as farmers, thus controlled or eradicated, albeit usually late in the disease spread process. Disease notification in this manner is called ‘passive surveillance’ [38]. Meanwhile, ‘active surveillance’ is a policy choice, an active procedure to detect a disease early so that an outbreak is manageable and damages can be avoided [39]. The question is how much to spend on active surveillance so that the total cost of controlling an incursion, along with total damages and the cost of detecting it early, is the smallest.

With this in mind, we formulate the surveillance problem as a two-stage stochastic programming problem. A decision has to be made on how much to spend on active surveillance in the first stage, after which the spread of an outbreak is known. This spread is independent of the first stage decision which is assumed to be fixed once it is made. Thus, our problem can be expressed in the form:

$$\begin{array}{c} \underset{\text{the}\;\text{minimum}\;\text{cost}}{\underset{︸}{c^{*}}}=\underset{q\in Q}{\text{min}}\;\{E[C(q,\xi )]\}=\underset{\text{active}\;\text{surveillance}\;\text{cost}}{\underset{︸}{C_{\text{as}}\left(q\right)}}+\underset{\text{expected}\;\text{outbreak}\;\text{cost}}{\underset{︸}{\lambda E[C_{\text{outbreak}}\left(q,\xi \right)]}} \end{array}$$ (1)

where *C*(*q*, *ξ*) is a cost function of *q* ∈ **Q**, a decision vector representing active surveillance efforts; *ξ* ∈ ***ξ***, is a multi-dimentional random vector associated with how an outbreak unfolds; and λ is an outbreak arrival rate which is assumed to be known.

The goal here is to find some policy *q* that is feasible for all the possible scenarios and minimizes the expectation E[C(q,ξ)]. However, as the number of outbreak scenarios can be very large when the spread is over both time and space, not to mention highly contingent upon the characteristics of the disease, directly computing E[C(q,ξ)] is likely to be infeasible for most surveillance problems. Therefore, we approximate the problem using SAA.

### 2.1 The sample average approximation method

SAA is a two-part method that uses sampling and deterministic optimisation [40]. The idea is to approximate E[C(q,ξ)] using sample average estimates which are derived from independently and identically distributed (iid) samples of *ξ*. For each scenario of *ξ*, the problem (1) is deterministic, and therefore, deterministic optimisation techniques can be applied. The process is repeated with many different scenarios and samples, and candidate solutions are tested and validated until convergence to the ‘true’ solution becomes clear.

Specifically, the implementation of SAA involves a three-stage procedure. In the first (training) stage, a lower bound for the objective function c* is estimated as:

$$\begin{array}{c} {\bar{c}}_{N}=\frac{1}{M}\sum_{m=1}^{M}c_{N}^{m} \end{array}$$ (2)

where $c_{N}^{1},c_{N}^{2},\ldots ,c_{N}^{M}$ are objective values obtained from *M* independently and identically distributed (iid) generated samples of size *N*. Associated with these objective values are candidate policy solutions ${\hat{q}}^{1},{\hat{q}}^{2},\ldots ,{\hat{q}}^{M}$. In the second (testing) stage, an iid sample of size *N*′, typically being much bigger than *N* and generated independently from previous samples is used to estimate an upper bound for c* for any feasible point $\hat{q}\in Q$ by:

$$\begin{array}{c} {\hat{c}}_{N^{\prime }}\left(\hat{q}\right)=\frac{1}{N^{\prime }}\sum_{n^{\prime }=1}^{N^{\prime }}C\left(\hat{q},\xi_{n^{\prime }}\right) \end{array}$$ (3)

Naturally, the best candidate solution ${\hat{q}}^{*}$ selected is the one that gives the smallest objective value ${\hat{c}}_{N^{\prime }}\left({\hat{q}}^{*}\right)$, or ${\hat{q}}^{*}\in \text{arg}\;\text{min}\left\{{\hat{c}}_{N^{\prime }}\left(\hat{q}\right):\hat{q}\in \left\{{\hat{q}}^{1},{\hat{q}}^{2},\ldots ,{\hat{q}}^{M}\right\}\right\}$. In the third (validating) stage, another iid sample *N*″, being also much larger than *N*, is generated independently from previous samples to check the quality of the solution by estimating the ‘optimality gap’ as:

$$\begin{array}{c} \text{gap}\left({\hat{q}}^{*}\right)={\hat{c}}_{N^{\prime \prime }}\left({\hat{q}}^{*}\right)-{\bar{c}}_{N} \end{array}$$ (4)

The three-stage procedure repeats with increasing samples *N*, until the **gap** $\left({\hat{q}}^{*}\right)$ is small enough to ensure convergence of the estimated solution to the true one [41]. Finally, the standard errors of SAA estimators in Eqs (2)–(4) are estimated using otherwise standard statistical methods (for relevant formulas, see [40]).

SAA has all of the desirable asymptotic statistical properties due to being underpinned by the ‘Law of Large Numbers’ (LLN), so that as sample size grows, the estimated mean gets closer to the ‘true’ average of the population. It particularly suits surveillance problems since it removes the need of specifying a functional form disease spread, and random factors are realised outside the optimisation routine (a so-called exterior sampling technique). Estimates of global optima are guaranteed if the set of decisions is convex and the objective function is convex in policy choices for all scenarios [42]. While the first assumption is likely in our case, the second is also expected since dangerous AIS/TAD spread swiftly and generally outpace the economies-of-scale, if any exist, of control measures.

Nonetheless, as indicated, the challenge faced by SAA applications is their computational expense. All scenarios are processed at once, in a batch manner, to find the optimum, so the procedure is memory-demanding. However, solution quality depends on the number of scenarios. This challenge explains the absence of SAA in the biosecurity literature, in which problems with time, space and randomness all being considered, forcing the need for an extremely large dimensional platform. In this context, we propose techniques, described in the following sub-sections, to manage the dimensionality of TAD surveillance problems so that SAA can be effectively applied.

### 2.2 Infection tree model

To facilitate the application of SAA, we design an infection tree with two key features. First, it allows multiple independent contacts between farms over time, resembling reality. Specifically, a farm can be the source of infection for several farms, and likewise, one farm can be infected by several farms. Second, the infection path evolves independently from policy choices. Here, each tree is an outbreak without any interventions, and it serves as a control case, to which different policies are applied and compared.

To demonstrate the infection tree’s advantage, we use a simple example with three farms, namely A, B and C. Between any two consecutive points in time, there are nine distinct contacts possible, including those made with themselves (e.g. A→A, A→B, A→C, B→A, B→B, etc.). Note that the direction of contacts is essential here and represented by an arrow →. Without loss of generality, at the outset (*t* = 0), suppose only farm C is infectious. We further assume that only two successful contacts C→A and B→C are made between *t* = 0 and *t* = 1. Consequently, at *t* = 1, A is newly-infected with the source of infection being C while C remains infectious. Contact from B→C, though successful, does not and will not affect the development of the infection tree, as B is not infectious. Therefore, there is no need to record this contact in the model to save memory. Moving to *t* = 2, suppose, only A→C, A→B, and C→B are successful. Thus B is newly-infected with two sources of infection, namely A and C, and all three farms are infectious at *t* = 2.

**Fig 1:**
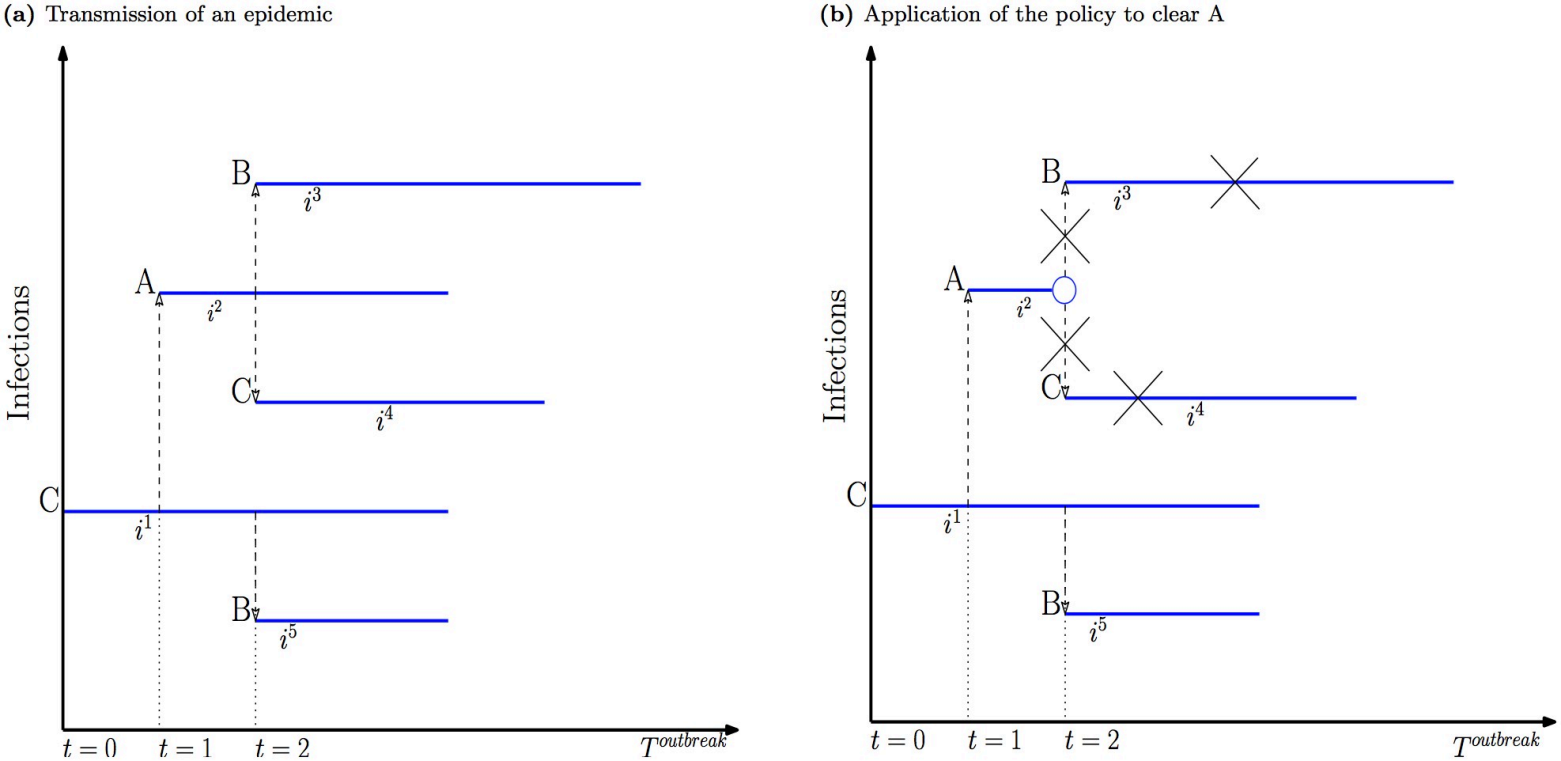
Infection tree model.

Our infection tree model keeps only contacts/infections made by infectious farms (i.e. Infectious → Susceptible and Infectious → Infectious). Thus, for this example, there are five infections, including the first one, in which C is the source of infection for itself. Infections are denoted as *i*_*n*_ where *n* = 1, 2, …, 5 indicate the order of infection time (the left panel of Fig 1). For each infection, four pieces of information are kept. They include the name/id of the infected farm which is located at the beginning of each solid line; the name/id of the source farm that spreads the infection, located at the beginning of each dashed-line; and the times the infected farm gets infected and removed, respectively, represented by the two endpoints of the solid line. To this end, the length of the solid line is the age of an infection.

The infection tree yields three benefits to the application of SAA. First, keeping infections with only the information needed for modelling is efficient in data storage. Second, independence among infections is key to efficient computing. It allows an infection tree to be pruned easily when different policies are applied. For example, as seen in the right panel of Fig 1, suppose a policy that clears A at *t* = 1 is applied. This results in a cut of the branch that grows from the infection that begins with A being infected at *t* = 1. This cut does not affect any other infections. Therefore, no computing effort is required to know whether B remains infectious at *t* = 2 since, in this case, the infection C→B remains intact. Finally, the infection tree facilitates tracing the source farm and finding the age of an infection, thus further easing computation. The latter is important to identify whether an infected farm reaches the point where it can be naturally detected or immunised.

**Fig 2:**
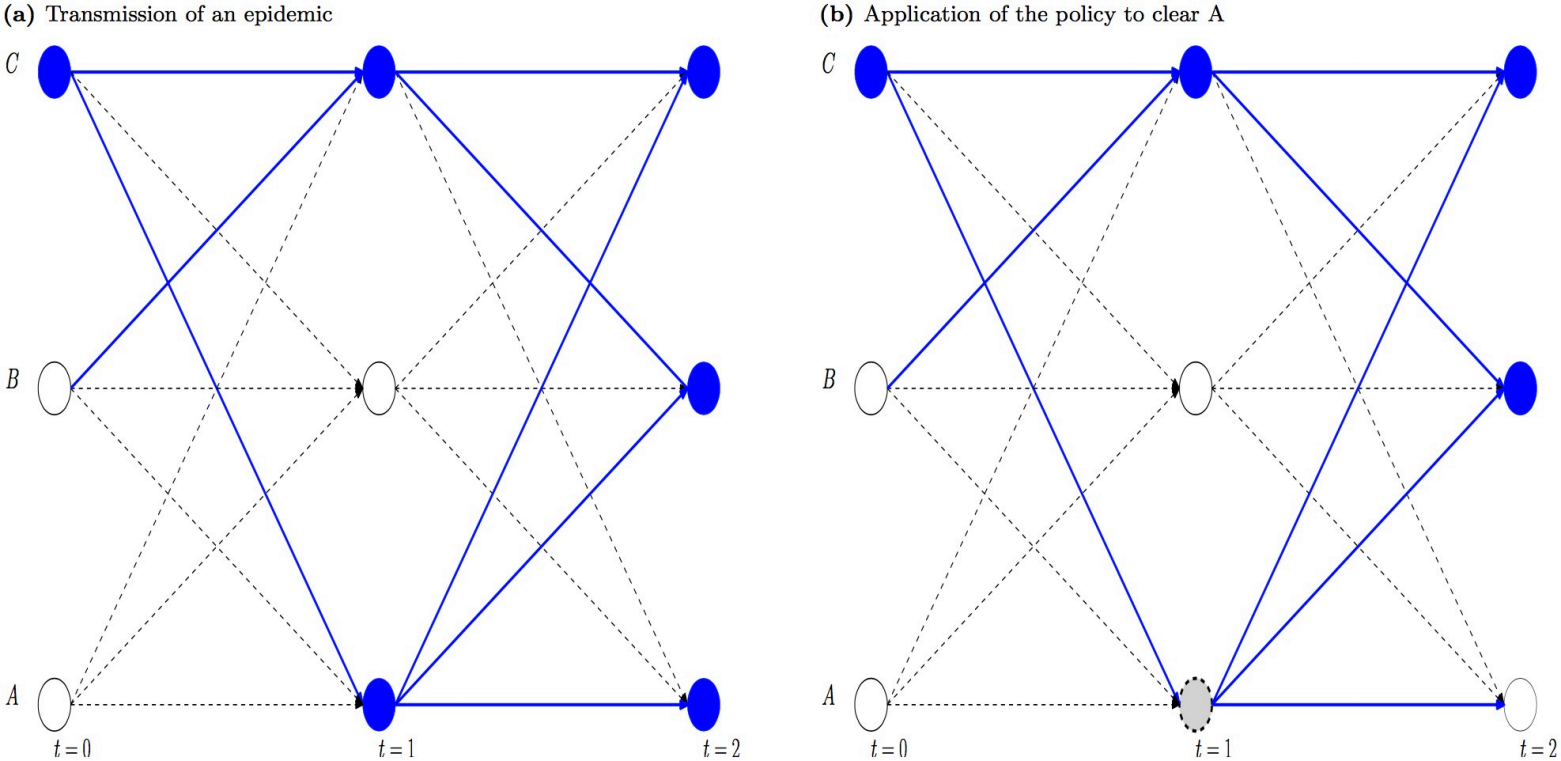
Network model.

The infection tree’s benefits might be better seen in comparison with a network model, a commonly-used tool to represent objects and their relationships [43]. The previous example is now presented in Fig 2 for illustration: the left panel shows solid balls as infected farms, solid lines as successful contacts, and dashed lines as probable but unsuccessful contacts, while the right panel shows the same case but with A being cleared. As can be seen, the network model has at least three problems, due mainly to its focus on farms and contacts. First, it needs considerable memory, at each time step, to keep the state of all farms and all successful contacts including B→C, which does not matter to the disease progression. Second, it requires large computational time to sort out the state of each farm once a policy is applied. The reason is that one farm can be infected by several source farms, but these infection paths are not kept separately. Specifically, B is infected even when A is removed, as B is infected by not only A but also C. In contrast to our infection tree model, the state of B in the network model is not known without performing a substantial computing exercise, which costs computational time and memory. Third, it is difficult to establish the infection age of each farm, which is vital for disease progression. In short, a network model is not amenable to surveillance problems of large dimension given its computational inefficiency.

### 2.3 Infection tree pruning rules

Despite their benefits, infection trees, when they grow large, can substantially slow down the SAA procedure. To enhance computational efficiency, we introduce five pruning rules to cut infections that are not possible or sensible in any tree, irrespective of policies applied. These rules are relevant to Susceptible-Infectious-Removed (SIR) models where animals are either removed or immunised after being infected.

**Fig 3:**
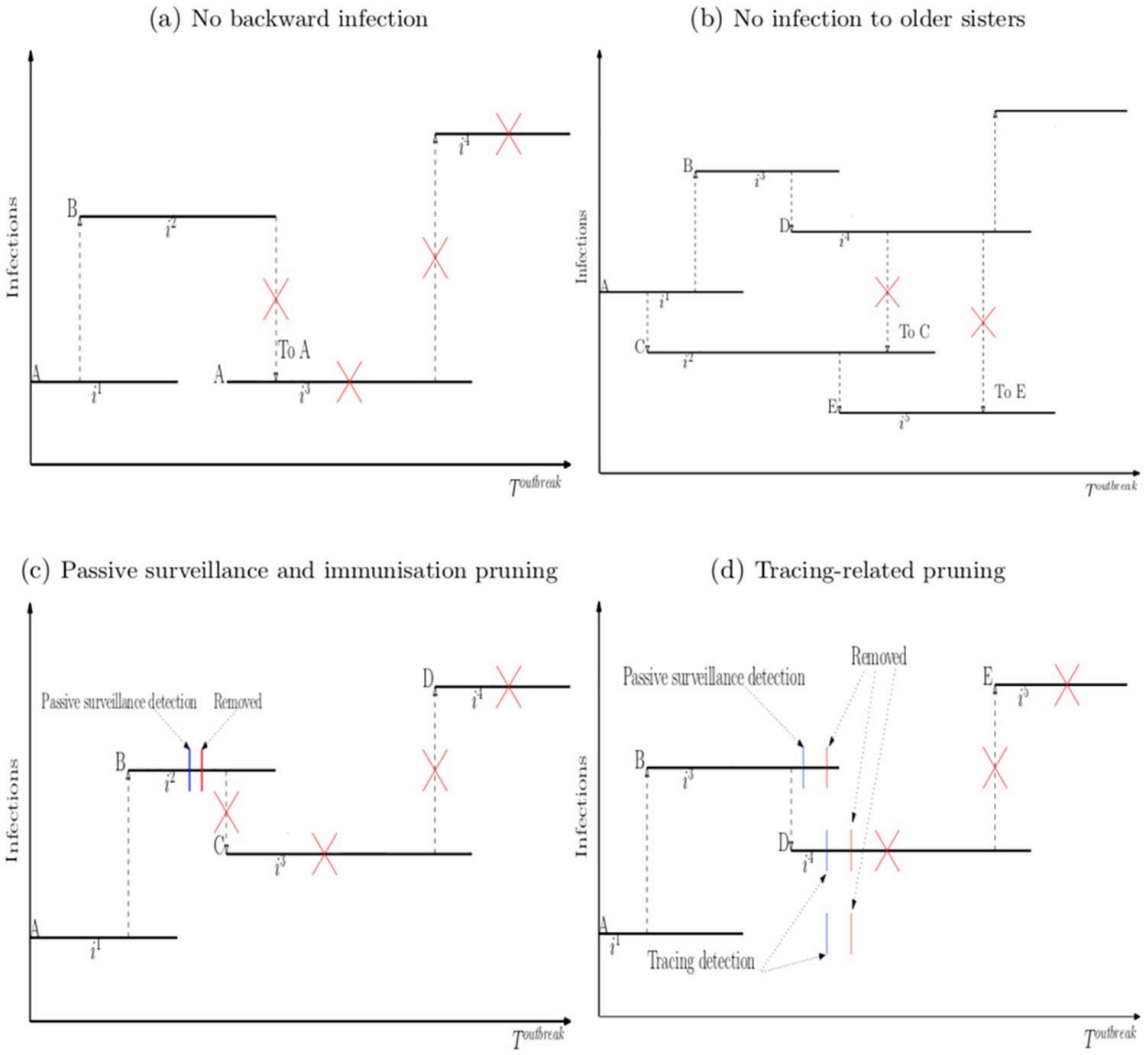
Infection tree pruning rules.

*Rule 1: No backward infection*. This rule trims infections that spread the disease back to their source farms. For example, in Fig 3, panel (a), infection *i*_2_ has A as the source farm of B. From B, any infections such as *i*_3_, in which B becomes the source farm of A, are not sensible. The reason is clear. Future contacts with A by other infected farms do not change the infection status of A since A is already infected. Furthermore, infection *i*_2_ which creates *i*_3_ would not exist without A being infectious in the first place. Therefore, infection *i*_3_ is not possible under any policies. Under this rule, it is cut from the tree together with its subsequent infections.
*Rule 2: No infection to older ‘sisters’*. This rule trims infections in which younger sisters spread the disease to older sisters. For example, in Fig 3, panel (b), infection *i*_1_ (mother) generates infection *i*_2_ (older child) and then infection *i*_3_ (younger child); later on, infection *i*_2_ creates infection *i*_5_ (grandchild) while infection *i*_3_ creates infection *i*_4_ (grandchild). In this case, *i*_3_, *i*_4_ and their ‘children’ are younger than *i*_2_, and hence are not able to generate *i*_2_ because *i*_2_ already exists when they are created.
*Rule 3: Passive surveillance and immunisation pruning*. An infection eventually ends with the infected farm either being detected by passive surveillance or by natural immunisation. If detected by passive surveillance, the infected farm will be removed. On the other hand, if naturally immunised, the infected farm will not be infectious. For that reason, Rule 3 trims all infections created by the ones detected by passive surveillance such as infection *i*_3_ in Fig 3, panel (c), and the ones that have infected farms being naturally immunised.
*Rule 4: Tracing-related pruning*. When an infected farm is detected by passive surveillance, there will be forward and backward tracing processes. Rule 4 trims all successful tracing cases. For example, in Fig 3, panel (d), infection *i*_3_ is detected by passive surveillance. For the backward-tracing, infection *i*_1_ which creates infection *i*_3_ will be trimmed if it is successfully traced. For the forward-tracing, on the contrary, any children of *i*_3_ and their subsequent infections will be cut with certainty, since *i*_3_, once detected and removed, is no longer able to create them.
*Rule 5: No long-distance infections after the first TAD detection*. The application of this rule is country-specific. Successful detection of TAD generally leads to a national livestock standstill, or movement restrictions, at least, followed by quarantine and severe movement controls [44]. Consequently, Rule 5 trims long-distance infections and their subsequent infections after the first TAD detection by passive surveillance is made.

### 2.4 Parallel processing

Parallel processing makes large-sized problems feasible in an SAA application. It can be applied to both simulation and optimisation routines. Various worker-processors can be used simultaneously to generate many large samples and calculate sample averages as specified in Eqs (2) and (3). Furthermore, parallel processing also plays an essential role in an SAA optimisation process, which is based on a direct search for the optimal point. Accordingly, in stage two (testing), objective function values for various policies applied to a large sample *N*′ of infection trees can be calculated simultaneously by many worker-processors. These worker-processors will then send their outputs to a master-processor to determine what is optimal. In the same fashion, Eq (4) can be executed with each worker-processor computing various upper bound estimates. These estimates are then sent to the master-processor to estimate the optimality gap. In short, many dimensionality issues in SAA applications can be largely addressed by parallel processing.

## 3 Case study: Optimal active surveillance against foot-and-mouth disease in the state of Victoria, Australia

Although generally useful, our proposed methods are applied here to the problem of optimising BMT-based active surveillance against FMD in Victoria during an epidemic or post-incursion. Specifically, we focus on exploring the potential use of BMT as an active surveillance tool when FMD has been found in the environment—an exercise highly policy-relevant to FMD-free countries in general and Australia, in particular, should an incursion occur. The surveillance problem is as specified in Eq (1), but with the arrival rate λ = 1. For this problem, our recent study finds that the optimal application of BMT is every day [33]. Our application aims to investigate whether this result holds with a better optimisation approach, in which time, space and randomness are fully considered.

### 3.1 Foot-and-mouth disease, active surveillance measure and the study area

FMD is one of the most dangerous TADs. It affects cloven-hoofed animals by causing debilitating effects such as weight loss, decrease in milk production and mortality in young animals [45]. Of the most concern is the ability of the FMD virus to survive in different environments for a long time and spread via various pathways [44–46]. Given its dangers, FMD ranks high in the list of notifiable animal diseases by the World Organisation for Animal Health (OIE), thereby causing substantial trade barriers to endemic countries, which now account for two-thirds of the world [1]. In spite of implementing stringent prevention measures, FMD-free countries remain under constant threat of an FMD outbreak due to increasing animal mobility, tourism and travel [47]. Indeed, over the last 15 years, these otherwise FMD-free countries alone have lost roughly $US 25 billion due to FMD outbreaks [3].

From this perspective, increasing attention has been paid to enhancing local surveillance against FMD, in FMD-free countries, to ensure early detection and quicker return to the market. A few measures have been proposed for active surveillance [48–50]. However, none has been applied in practice to the best of our knowledge. In theory, bulk milk testing (BMT) seems a promising measure since it can be embedded in the existing system that mass-screens milk for common and indigenous diseases [38]. BMT uses a real-time reverse transcription-polymerase chain reaction (rRT-PCR) [51]. The idea of the test comes from a finding that the milk from FMD incubating cattle contains an FMD virus for up to 4 days before clinical signs of the disease become evident [52, 53]. Therefore, we consider BMT as an active surveillance measure for detecting FMD, operating on top of the existing passive surveillance system.

In terms of the study area, there are a few reasons to choose the state of Victoria in Australia. First, Australia is one of the world’s largest exporters of livestock [54]. Since its agricultural system is highly exposed to the world market, damages from a possible FMD outbreak are undoubtedly substantial [55, 56]. Second, Australia is exploring more active ways to enhance early detection of FMD, a system which generally relies solely on passive surveillance [57, 58]. Third, the state of Victoria is thought to bear the largest relative risk of an FMD introduction, establishment and spread [59]. The reason for this is straightforward. Victoria has the highest livestock and human population density in Australia, and its livestock production is relatively close to high volume air and sea ports. Its environmental conditions are also generally suitable for FMD virus survival. Furthermore, the state is vulnerable to a widespread outbreak due to the scattered distribution of pig farms which carry the biggest risk of being exposed to and infected by the FMD virus among all cloven-hoofed animals [57]. Finally, Victoria is the centre of Australia’s dairy production [60], making it the most suitable state for using BMT as an active surveillance measure.

**Fig 4:**
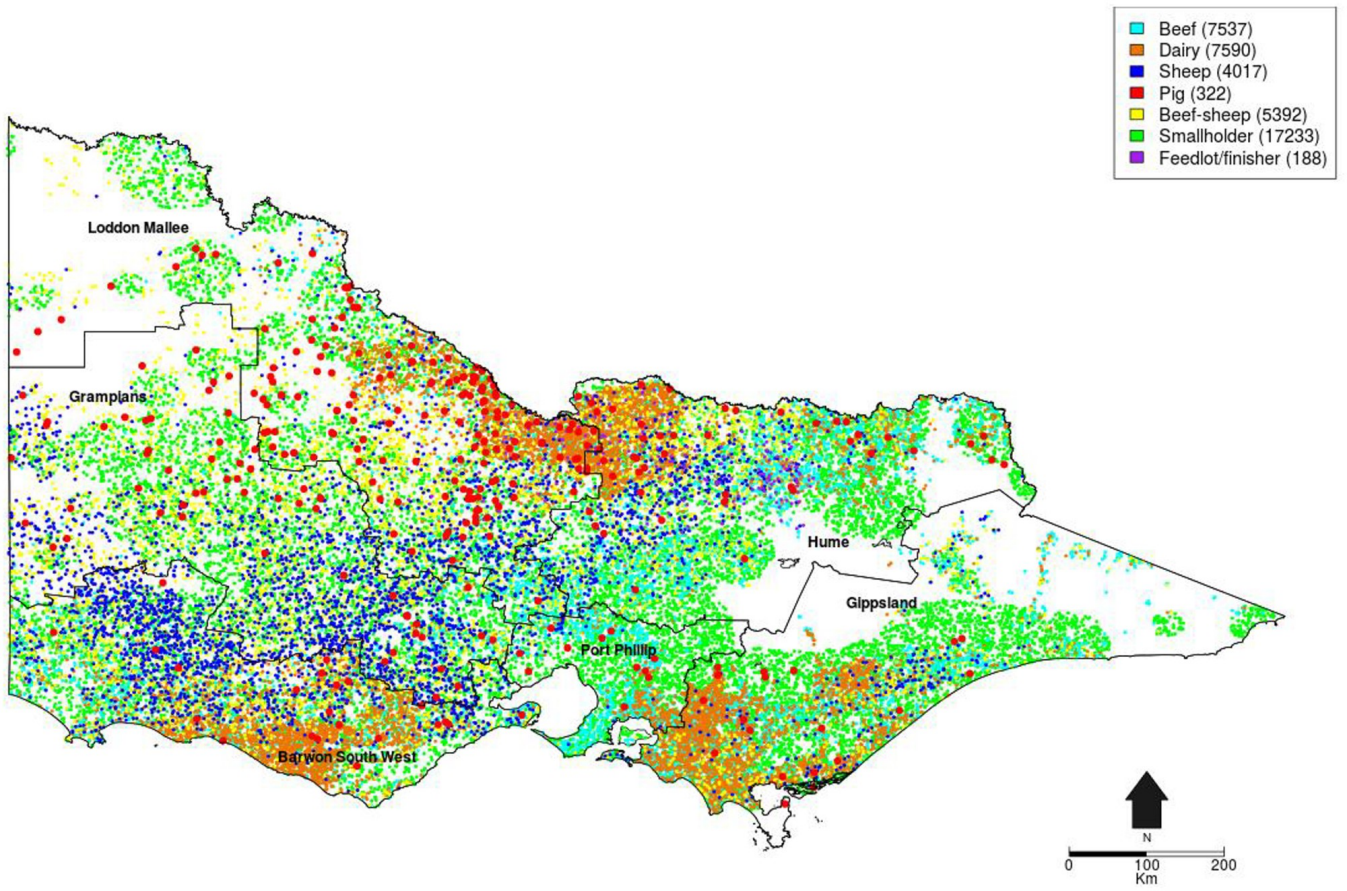
Farm distribution in Victoria.

As a state, Victoria has 42,279 farms in total. As seen in Fig 4, farms are classified into seven categories, including beef, dairy, sheep, pig, mixed beef-sheep, smallholder, and feedlot. However, even within in each category, farms are not homogeneous in size as well as livestock composition. In summary form, Victoria is home to 62% (1.6 million head), 22% (0.5 million head), 21% (14 million head) and 9% (2.1 million head) of Australia’s populations of dairy cattle, pigs, sheep and lambs, and meat cattle, respectively, while occupying just over 3% of Australia’s land mass [61].

### 3.2 FMD dispersal

In line with existing literature, our farms are classified as either *susceptible*, *infectious* or *removed*. Consider a large population of farms *F*. At the outset, a random farm gets FMD-infected from an outside source. This farm is called the *seed* farm which spreads the disease to other farms via successful contacts.

In addition to being random, the transmission between farms also depends on farm-level characteristics such as livestock size and type, and the distance between susceptible and infectious farms (e.g. [8, 62]). Accordingly, the probability *π*_*f*_ that a susceptible farm *f* becomes an infected one *f*′ in a given day is defined as:

$$\begin{array}{c} \begin{array}{ccc} \pi_{f} & = & 1-\text{exp}[-S^{T}P_{f}\sum_{f^{\prime }}\left(T^{T}P_{f^{\prime }}K+\delta \right)] \end{array} \end{array}$$ (5)

where *S*^*T*^ and *T*^*T*^ are the transposes of the *L* × 1 vectors of susceptibility (risk of catching the disease) and transmission (rate of spreading the disease), respectively, associated with livestock type *i*, where *i* ∈ *L* = {*cattle*, *pig*/*sheep*, *others*}; *P* is the farm-level livestock size *L* × 1 vector; and *K* ∈ **K** is the dispersal kernel *F* × *F* matrix with its elements determined by a three-parameter function, decreasing in the distance *d*_*f*,*f*′_ between susceptible and infectious farms [63], in the form:

$$\begin{array}{c} \begin{array}{ccc} K(d_{f,f^{\prime }}) & = & \frac{k_{o}}{1+{\left(d_{f,f^{\prime }}/d_{0}\right)}^{\alpha }} \end{array} \end{array}$$

and *δ* explains some random factors, especially long distance jumps which are not well explained by *S*, *T* and *K* [64].

### 3.3 The infection tree

FMD transmission is a stochastic spatial dynamic process, for which each realisation forms an infection tree or an outbreak scenario. Stochastic factors can be grouped under two categories: those related to the creation of an infection in the tree and those determining how an infection ends. The first group gives information on names/id of the source and the infected farms, as well as the time an infection begins (*t*_*b*_) while the second group lets us know when an infection ends (*t*_*e*_).

The creation of an infection is governed by Eq (5). It is worth noting that there is a latent period (*τ*_latent_) when an infection begins, during which the farm is not infectious [65]. We take this period into account in our computation.

Meanwhile, an infection can end in one of the three ways. First, an infected farm can be detected and removed by passive surveillance after *τ*_ps_ days, with a probability *p*_ps_, which depends on farm type. Alternatively, if BMT-based active surveillance is applied, *t*_*e*_ will depend on not only *τ*_ps_ and *p*_ps_, but also the BMT frequency *q*. Second, an infected farm can be detected and removed via tracing activities after a tracing period of *τ*_trace_. Finally, an infected farm can be naturally immunised and becomes non-infectious after a clinical period of *τ*_im_, which also depends on the farm type.

To describe the disease dynamics, we first introduce some notations. We denote $x_{t}^{j}$ as the infection state of farm *j* at time *t* where *j* ∈ *F*. There are two values in $x_{t}^{j}:x_{t}^{j}=0$ means the farm is susceptible (i.e., being a farm of type *f*), while $x_{t}^{j}=1$ means the farm is infectious (i.e., being a farm of type *f*′). We denote $y_{t}^{j}$ as the control state of farm *j* at time *t*, with two values: $y_{t}^{j}=1$ means the infection at farm *j* ends due to being detected through surveillance or by tracing activities; otherwise, $y_{t}^{j}=0$. We denote $g_{t}^{j}$ as the immunisation state of farm *j* at time *t*, with two values: $g_{t}^{j}=1$ means the infection at farm *j* ends by natural immunisation; otherwise, $g_{t}^{j}=0$. Finally, we denote *X*_*t*_, *Y*_*t*_ and *G*_*t*_ as *F* × 1 vectors of infection, control and immunisation states of all farms at time *t*.

Initially (i.e., *t* = 0), vector *X*_0_ has all elements equal to zero with one exception for the seed farm, being randomly drawn from the pig farms, the most likely case according to [58]. Meanwhile, vectors *Y*_0_ = 0 and *G*_0_ = 0. That is, at the outset, the infected seed farm is not at *t*_*e*_ for any reason while the remaining farms are all susceptible.

Moving forward in time, the infection state of each farm $x_{t}^{j}$ at *t* where *t* > 0 depends on four factors. They include (i) its probability $\pi_{t}^{j}$ of getting infected at *t* as governed by Eq (5); (ii) its infection state in the previous time period $x_{t-1}^{j}$; and (iii) its control state $y_{t}^{j}$ and (iv) immunisation state $g_{t}^{j}$. The value of $x_{t}^{j}$ thus can be determined using:

$$\begin{array}{c} \begin{array}{ccc} x_{t}^{j} & = & \left\{ \begin{array}{c} 1\;\;\text{if}\;x_{t-1}^{j}+\pi_{t}^{j}-2\left(y_{t}^{j}+g_{t}^{j}\right)\geq 1\\ 0\;\;\text{otherwise} \end{array}\;\text{for}\;\;j\in F,t\in \left[0,T^{\text{outbreak}}\right]\right. \end{array} \end{array}$$ (6)

where *T*^outbreak^ is the end point of an outbreak which depends on how an outbreak unfolds and the effectiveness of passive and active surveillance; the control and immunisation states of each farm, $y_{t}^{j}$ and $g_{t}^{j}$ at *t* which also depend on their states in the previous time period and the realisation of random factors as follows:

$$\begin{array}{c} \begin{array}{ccc} y_{t}^{j} & = & \left\{ \begin{array}{c} 1\;\;\text{if}\;y_{t-1}^{j}+\varepsilon_{t}^{j_{y}}\geq 1\\ 0\;\;\text{otherwise} \end{array}\;\text{for}\;\;j\in F,t\in \left[0,T^{\text{outbreak}}\right]\right.\\ g_{t}^{j} & = & \left\{ \begin{array}{c} 1\;\;\text{if}\;g_{t-1}^{j}+\varepsilon_{t}^{j_{g}}\geq 1\\ 0\;\;\text{otherwise} \end{array}\;\text{for}\;\;j\in F,t\in \left[0,T^{\text{outbreak}}\right]\right. \end{array} \end{array}$$ (7)

where $\varepsilon_{t}^{j_{y}}$ and $\varepsilon_{t}^{j_{g}}$ depend on how an infection ends (as described earlier) and the states of other farms in the previous period. It is worth noting that $y_{t}^{j}$ and $g_{t}^{j}$ are mutually exclusive, i.e., if $y_{t}^{j}=1$ first, then $g_{t,\ldots ,T^{\text{outbreak}}}^{j}=0$, and vice versa.

### 3.4 Economic costs

We aim to find the optimal surveillance policy ${\hat{q}}^{*}$ that minimizes the approximate E[C(q,ξ)], specified in Eq (1). To do so, we need to calculate *C*(*q*, *ξ*) for each infection tree and each value of *q*. That is, each tree is first built without any policies and as a scenario of *ξ* which comprises random factors described in sub-section 3.3. Each policy of *q* is then applied to the tree to trim it, and to find the corresponding value of *C*(*q*, *ξ*).

In a typical surveillance problem, *C*(*q*, *ξ*) has four components, including (i) the cost of active surveillance *C*_as_; (ii) revenue loss *C*_r_; (iii) outbreak management cost *C*_m_; and (iv) the cost of eradication *C*_e_, for which the last three cost components amount to outbreak cost in Eq (1).

Our estimation of *C*_as_ follows Garner et al. [58] and Kompas et al. [33]. Accordingly, a tanker is assumed to visit *h* farms in one trip to collect milk every day, and *q* is the testing interval of BMT (i.e., one test per *q* day(s)). The BMT-based *C*_as_ is calculated as:

$$\begin{array}{c} \begin{array}{ccc} C_{\text{as}} & = & c^{\text{bmt}}\times \frac{M_{\text{df}}}{q\times h}\times D_{\text{outbreak}}+E_{\text{one-off}}\times M_{\text{fac}} \end{array} \end{array}$$ (8)

where *c*^bmt^ is the unit cost per rRT-PCR milk test; *M*_df_ is the number of dairy farms; *D*_outbreak_ is the outbreak duration starting from the time FMD is detected by passive surveillance, plus the time for culling, and the time for quarantine, minus the time for setting up BMT testing equipment; *E*_one-off_ is the one-off cost of the testing equipment; and *M*_fac_ is the number of milk collection points or factories in Victoria.

The revenue losses are largely caused by immediate and prolonged bans of exports to Australia’s FMD-sensitive markets and depressed domestic prices [56]. The impact of an FMD outbreak on revenues can be long-lasting, and is largest in the first year [55]. Therefore, the revenue losses are calculated as:

$$\begin{array}{c} \begin{array}{ccc} C_{\text{r}} & = & c_{\text{r1}}\left(D_{\text{outbreak}}+D_{\text{mkt1}}\right)+c_{\text{r2}}D_{\text{mkt2}} \end{array} \end{array}$$ (9)

where *c*_r1_ and *c*_r2_ are the daily revenue losses in the first and the following years, respectively; *D*_outbreak_ is outbreak duration starting from the time FMD is detected by passive surveillance, plus the time for culling, *τ*_cull_, and the time for quarantine, *τ*_quar_; *D*_mkt1_ and *D*_mkt2_ are the corresponding durations when markets react to an FMD outbreak, inducing revenue losses.

The cost of outbreak management is calculated as:

$$\begin{array}{c} \begin{array}{ccc} C_{\text{m}} & = & c_{\text{m}}\times D_{\text{outbreak}} \end{array} \end{array}$$ (10)

where *c*_m_ is the daily operating cost of a FMD disease control centre(s).

Finally, the cost of eradication which includes expenses on compensation to farms, slaughtering and disposal, as well as decontamination [66–68] is calculated as:

$$\begin{array}{c} \begin{array}{ccc} C_{e}=\sum_{t=0}^{T}\sum_{j}c_{e}^{j}(\begin{array}{c} y^{j}\\ w^{j} \end{array})\; & \text{for} & j\in F \end{array} \end{array}$$ (11)

where $c_{e}^{j}$ is a row vector of the farm specific eradication cost, *y*^*j*^ is defined earlier, and *w*^*j*^ is a vector of farms culled as a preemptive measure, which is typically dependent on the controlled farm (*y*^*j*^).

### 3.5 Parameterisation

FMD susceptibility and transmissibility coefficients in Eq (5) are estimated using 200 simulations by the AusSpread—the FMD spread model for Australia [28]. AusSpread is built as a susceptible-latent-infected-recovered (SLIR) model. Its input is farm point-location data with detailed information on herd size, animal and production types, among others. It can simulate the spread of disease by way of animal movements through saleyards, wind-borne spread, and local spread, as well as by direct and indirect farm-to-farm contact. The model simulation outcomes are a series of random iterations, thus forming a set of random data, which can be used to estimate parameters for an epidemic. For this application, all estimates are statistically significant at 1% level and have expected signs (Table 1).

**Table 1:** Foot-and-mouth disease susceptibility and transmissibility coefficient estimates.

Coefficient	Description	Cattle	Pig/Sheep	Other livestock type
S	Susceptibility coefficient	1	0.0524*	0.0427*
		(0)	(0.0033)	(0.0027)
T	Transmissibility coefficient	0.446*	0.0354*	0.00367*
		(0.021)	(0.0040)	(0.0139)
*k*_0_	Kernel function parameter	1	1	1
		0	0	0
*d*_0_	Kernel function parameter	1.571*	1.571*	1.571*
		(0.041)	(0.041)	(0.041)
*α*	Kernel function parameter	3.61*	3.61*	3.61*
		(0.103)	(0.103)	(0.103)
*δ*	Long distance jump coefficient	0.00000870*	0.00000870*	0.00000870*
		(0.000000402)	(0.000000402)	(0.000000402)

Estimation based on an average outbreak in 200 simulated outbreaks from the AusSpread model. Parameter values of *S* for cattle and *k*_0_ are fixed at one to avoid having an undefined problem, in which the number of equations are fewer than the number of unknowns. Standard errors are reported in parenthesis. (*): statistically significant at the 1% level. In Auspread, a farm can not be infected twice in an outbreak (no further infection to the same farm), therefore we adopt an adjustment coefficient (approximately 50% higher) to the (estimated) infection probability to better accommodate our infection tree modelling context.

Values for other epidemiological parameters are from existing literature or the AusSpread model (Table 2). In particular, the FMD latent period *τ*_latent_ is 4 days according to [65]. The time that FMD can be detected by passive surveillance varies by farm type. The probability of FMD being detected by passive surveillance, or *p*_ps_, is the product of the reporting probability by owner/manager which also depends on farm type, ranging from 0.005 to 0.836, and the probability of their reports being adequately investigated which is equal 0.59 for all farms^1^ Author’s assumption and estimate based on AusSpread model. Based on the AusSpread model, the culling time *τ*_cull_ is from 1 to 3 days, depending on farm type, while other parameter values such as the tracing time *τ*_trace_ (3 days), the quarantine time *τ*_quar_ (90 days), and the pre-emptive culling applied to direct contacts, which are random and governed by Eq (5). Finally, infected farms become naturally immunised after *τ*_imm_ of 28 to 32 days, conditional on farm type.

**Table 2:** Parameter values and description.

Parameter	Description	I	II	III	IV	V	VI	VII	Unit
*τ*_latent_	Latent period^(*a*)^	4	4	4	4	4	4	4	day
*τ*_ps_	Time from becoming infectious to reaching 20% clinical prevalence^(*c*)^ + *τ*_latent_	13+4	11+4	11+4	12+4	20+4	16+4	10+4	day
*p*_ps_	Passive surveillance detecting probability^(*c*)^	0.0602	0.3723	0.4932	0.5216	0.0030	0.0407	0.0454	
*τ*_im_	Duration of clinical period above 20% clinical prevalence threshold^(*c*)^ + *τ*_ps_	16+17	16+15	15+15	14+16	8+24	12+20	14+14	day
*τ*_trace_	Tracing time^(*c*)^	3	3	3	3	3	3	3	day
*τ*_cull_	Culling time^(*c*)^	2	3	1	3	2	2	1	day
	Unit cost per farm slaughtering, disposal and decontamination^(*d*)^	49,322	218,428	70,461	91,599	49,322	49,322	8,103	$
		For the whole outbreak							
*c*^bmt^	Unit cost per bulk milk test^(*e*)^	36							$
*c*_m_	Daily operating cost of an FMD disease control centre(s)^(*f*)^	0.475							$ Mil
*c*_r1_	Daily revenue loss in the first year^(*b*)^	14.8							$ Mil
*c*_r2_	Daily revenue loss in the 9 following year^(*b*)^	0.246							$ Mil
*τ*_quar_	Quarantine time^(*d*)^	90							day
		For the whole outbreak							Unit
*h*	Number of farms visited by a milk tanker in one trip^(*e*)^	5							farm
*τ*_equip_	Testing equipment set-up time^(*e*)^	7							day
*M*_df_	Number of dairy farms^(*e*)^	7,590							farm
*E*_one-off_	One-off cost of testing equipment^(*e*)^	500,000							$
*M*_fac_	Number of milk factories^(*e*)^	25							factory

Farm types: I (Beef); II (Beef feedlot); III (Dairy); IV (Pig); V (Sheep); VI (Mixed sheep/beef); VII (Smallholder); All values are in Australian Dollar 2014; (a): [65]; (b): [55] and [56]. (c): Author’s assumption and estimate based on AusSpread model; (d): Calculated from [69]; For compensation, unit values per animal type are: $802 per cattle in beef farms and cow in dairy farms; $75 per sheep; $240 per pig; $ 942 per cattle in beef feedlot farms; and $372 per animal in other farm types. (e): [58] and [33]; (f): Calculated based on [70] and [69];

As for economic costs (Table 2), Garner et al. [58] and Kompas et al. [33] discuss in detail the possibility of implementing BMT and its costing in Australia, noting that BMT is not yet commercially available. We follow their assumptions and estimates. Specifically, a typical milk tanker of 20,000 litres can collect milk from about five farms since the average size of an Australian dairy herd is 225 cows and the average yield is 17 litres/cow/day (i.e. 17 × 225 × 5≈20,000 litres). Thus there will be 552,552 milk samples to test on a daily basis for 7590 dairy farms in Victoria (i.e. (7590 farms/5) × 52(weeks) × 7(days)). It is required that two to four infected cows per farm and at least one infected farm contributing to a tanker for the test to have analytical sensitivityof 10^−3^ to 10^−2.5^ (i.e. 2 × 17 litre to 4 × 17 litre/20,000 litre ≈ 10^−3^ to 10^−2.5^). We use the farm level threshold of three infected cows for detection (∼10^−2.6^), which gives 95% diagnostic sensitivity of milk rRT-PCR. We assume a delay of two days from when milk is tested until FMD is confirmed to allow for the traceback of individual farms and confirmation on investigations and testing. The efficacy of bulk milk testing is not sensitive to the testing interval (Garner et al. [58] and Kompas et al. [33]).

The eradication cost is farm-specific. It comprises: (a) compensation to farms based on the value of their livestock, and (b) slaughtering, disposal and decontamination expenses. Compensation is the product of the unit price from [69] and the quantity of each animal type in each farm. In contrast, estimates of slaughtering, disposal and decontamination expenses are by farm type as given by [69].

Meanwhile, daily revenue losses are applied to the whole outbreak. They are estimated based on the revenue losses due to an FMD outbreak of $5.4 and $0.807 billion in the first year and for the following 9 years, respectively. The cost breakdown for average revenue losses of $6.21 billion for a small FMD outbreak in Victoria are obtained based on a control strategy using a ‘stamp-out’ policy [56], and the assumption of 87% of these revenue losses being incurred in the first year [55]. Finally, the cost of BMT is from [33] and [58] while the daily operating cost of an FMD disease control centre is based on [69] and [70].

### 3.6 Results

Application of the SAA method to our case study is highly computer-intensive. In terms of required code and software, we use Fortran, C and R. For parallel processing, we use 24 processes over 3 quad core CPU computers with Hyper-Threading. The possible simulation numbers in our computational platform is thus 24 times larger than that in a similar uni-processing process. As shown in Table 3, when repeating the three-stage procedure of SAA, we increase the sample size *N* until the optimal gaps are stabilised at less than 0.5% (*N* → 4320), while keeping *M*, the number of samples, constant at 50. As a result, the number of simulations in the first stage increases up to 216,000 simulations. In the second and third stages, the sample sizes *N*′ and *N*″, in order to find the candidate optimal solution and check its quality remain constant at 144,000. Algorithms used for our computation are available upon request.

**Table 3:** Estimated outbreak costs with bulk milk testing implemented on top of passive surveillance and optimality gaps.

Estimates of	Trade losses not considered (at the estimated optimal BMT frequency ${\hat{q}}^{*}=5$)								
Outbreak cost without BMT	67.48	67.47	67.52	67.49	62.51	67.4	67.41	67.41	67.58
	(0.0294)	(0.0294)	(0.0294)	(0.0294)	(0.0294)	(0.0294)	(0.0294)	(0.0293)	(0.0294)
Lower bound (A)	62.54	62.46	62.62	62.73	62.86	62.79	62.72	62.67	62.70
	(0.164)	(0.102)	(0.079)	(0.071)	(0.072)	(0.054)	(0.059)	(0.050)	(0.044)
Upper bound (B)	62.74	62.69	62.72	62.71	62.74	62.66	62.67	62.68	62.77
	(0.0446)	(0.0445)	(0.0446)	(0.0445)	(0.0446)	(0.0446)	(0.0444)	(0.0444)	(0.0446)
Optimality gap (C = B-A)	0.200	0.230	0.100	-0.020	-0.120	-0.130	-0.050	0.010	0.070
	(0.209)	(0.147)	(0.124)	(0.115)	(0.116)	(0.099)	(0.103)	(0.095)	(0.089)
Percentage of the lower bound D = (C/A)*100%	0.32%	0.37%	0.16%	-0.03%	-0.19%	-0.21%	-0.08%	0.02%	0.11%
	Trade losses considered (at the estimated optimal BMT frequency ${\hat{q}}^{*}=2$)								
Outbreak cost without BMT	6277	6278	6278	6278	6278	6276	6277	6277	6279
	(12.428)	(12.430)	(12.430)	(12.431)	(12.430)	(12.426)	(12.428)	(12.428)	(12.433)
Lower bound (A)	6168	6171	6169	6170	6171	6171	6171	6170	6170
	(1.98)	(1.30)	(1.03)	(1.00)	(0.89)	(0.74)	(0.75)	(0.65)	(0.54)
Upper bound (B)	6170	6170	6170	6171	6171	6170	6170	6170	6171
	(0.567)	(0.567)	(0.566)	(0.568)	(0.568)	(0.568)	(0.566)	(0.567)	(0.570)
Optimality gap (C = B-A)	2.35	-0.43	1.41	0.37	-0.63	-1.58	-0.77	0.91	0.96
	(2.55)	(1.87)	(1.59)	(1.57)	(1.46)	(1.31)	(1.32)	(1.22)	(1.11)
Percentage of the lower bound D = (C/A)*100%	0.04%	-0.01%	0.02%	0.01%	-0.01%	-0.03%	-0.01%	0.01%	0.02%
N	480	960	1440	1920	2400	2880	3360	3840	4320

M = 50; N′ = 144,000; N″ = 144,000; Number of processes = 24. Value in AUD Million (2014). Standard errors are reported in parenthesis. All estimates are significant at 1% level except for the ones of the optimal gap which are statistically insignificant at 10% level.

**Fig 5:**
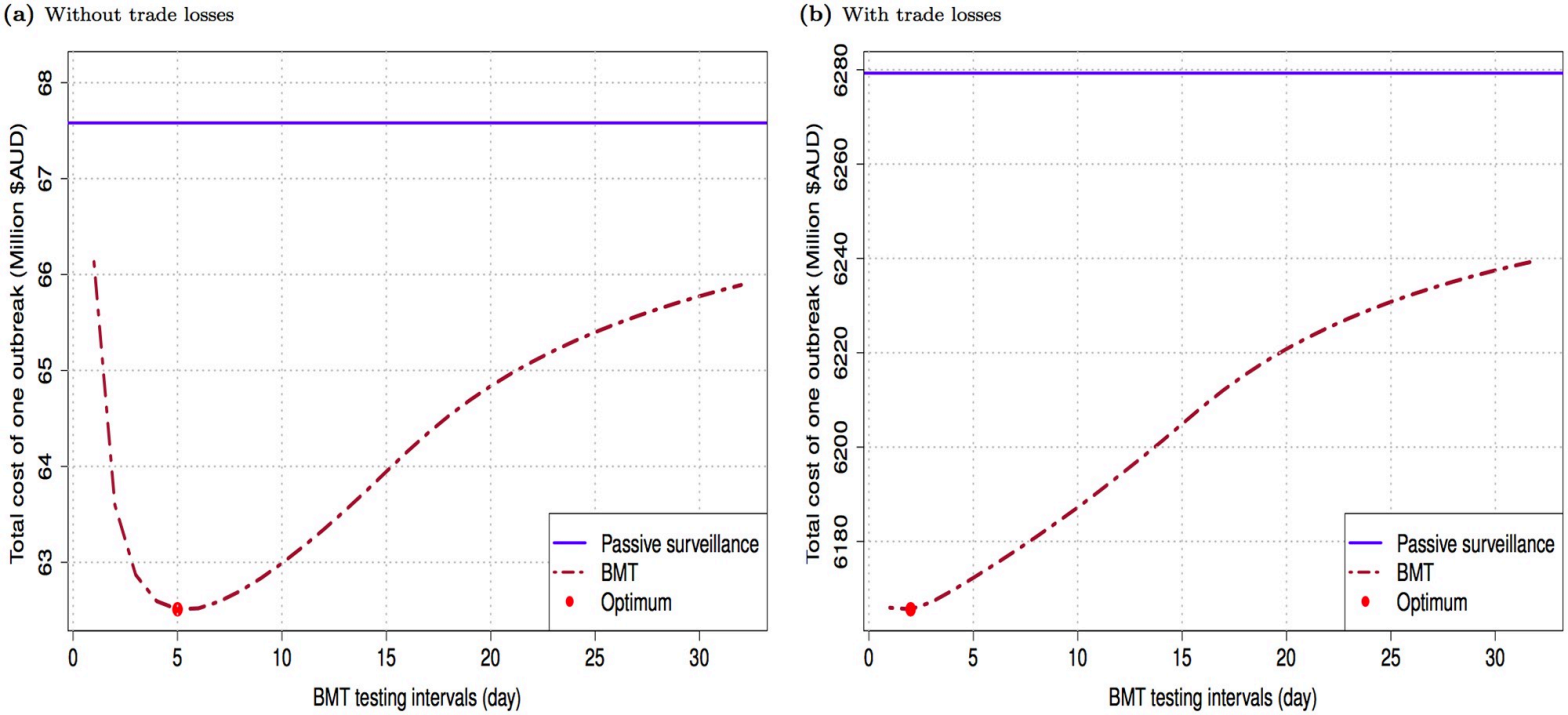
Total cost of an FMD outbreak versus BMT testing intervals.

We present results for both cases, with and without trade losses. We do so because trade losses, the much more likely case with a disease outbreak, are substantial and tend to dominate other variables while at the same time they can be hard to quantify precisely. The estimated optimal BMT testing interval ${\hat{q}}^{*}$ found for the case with trade losses is 2 days while that without trade losses is 5 days. These results are illustrated in Fig 5 using a sample of 144,000 infection trees. A clear trade-off is seen between active surveillance effort and the cost of outbreak control in both cases: without trade losses (panel (a)), and with trade loss (panel (b)). It is worth noting that in both cases, the minimized cost is much smaller than that of the control case which relies entirely on passive surveillance, thus making BMT-based active surveillance an economically-efficient policy.

Estimated outbreak costs with BMT-based active surveillance are presented in Table 3. The quality of these results is illustrated in the estimated optimality gaps. For brevity, we only present the results in the range that shows the convergence of the estimated solutions (i.e., the estimated optimality gap is not statistically significant from zero at any conventional level). The outbreak cost without BMT is about $6.28 billion and $67 million AUD with and without trade losses considered, respectively. The net gains of implementing BMT, which are the difference between the outbreak costs with and without BMT, are about $100 million and $5 million and AUD with and without trade losses considered. It is important to note, again, that an outbreak would generally result in trade losses (even if it is a small outbreak and relatively quickly eliminated).

Finally, we compare our results with those using the equation-based optimisation approach. Only the case when trade losses are considered is available for comparison. Our estimated optimal BMT frequency is now 2 days against 1 day found previously [33]. This result implies a saving of about $2.71 million AUD in active surveillance investment per outbreak. Our estimated outbreak costs are also lower, in the magnitude of $70–80 million AUD. These gains come at the cost of needing a more complex computational setting, but using the SAA approach and trimming methods outlined above makes the problem readily tractable. With the added use of a parallel processing routine, the results can be obtained in a few hours of computational time.

## 4 Discussion

IAS and TADs are incredibly harmful to the economy. The risk of their incursion has been on the rise due to rapid globalisation and increasing mobility in the world over the past few decades. This trend, coupled with the lack of perfect prevention measures, has made local surveillance against IAS/TAD an important instrument in many national biosecurity strategies. However, identifying an optimal level of surveillance against IAS/TAD is challenging due to the complexity of the problem and the fact that time, space and randomness in IAS/TAD transmissions need to be fully and explicitly considered. As a result, research and optimal economic results for early detection in this setting have not been available. This limits the best-practice biosecurity policy.

To narrow this gap, this paper has proposed some new techniques to circumvent the curse of dimensionality in this class of problems. In particular, we design an infection-independent tree model and use it in combination with pruning methods and parallel processing techniques to apply an SAA approach to a problem which otherwise would be too dimensionally large to solve. We demonstrate our model and techniques in identifying optimal active surveillance against FMD using BMT as an active surveillance protocol among more than 40,000 farms in Victoria, Australia, when uncertainty and spatial dynamics are taken fully into account. To this end, our application shows the considerable potential of using SAA, which is basically absent in economic literature, to solve biosecurity problems.

We find that it is optimal to implement BMT every two and five days with and without trade losses considered, respectively. The expected net gain of implementing BMT in these cases is 100 million and 5 million AUD, respectively. Compared with existing literature, our estimated optimal BMT frequency saves about $2.71 million AUD in active surveillance expenses per outbreak, with outbreak costs much lower at $70–$80 million AUD. The methodology proposed in this paper, which takes into full account all of the factors relevant to TAD spread dynamics, clearly enhances the precision of the analysis. We believe that this methodology is especially useful for biosecurity decision-making, where cost-effective economic measures are essential.

We envisage at least a couple of possible ways in which our work can be further improved in the future to aid policy choices for practical problems. The first and possibly the most important one is to allow surveillance policy to vary across the planning horizon. This decision-making process, albeit common in practice, is hardly addressed in the biosecurity literature due to the challenge of solving an unusually large multi-stage spatial-temporal, stochastic dynamic programming problem. To date, it has only been investigated in a single previous study, albeit in a deterministic setting and with a small range of spatial heterogeneity [22]. The second extension is to account for farm-level strategies that could potentially change the IAS/TAD spread. A mechanism of between-farm interaction has been suggested [71], but this is yet to be incorporated into an optimization surveillance model. With innovative modelling and efficient computational techniques, coupled with more computational power, future research could include richer variations in the decision-making process, while retaining the fundamentally spatial-temporal and stochastic nature of the problem.

## Supporting information

S1 Data (ZIP)
